# Quality control requirements for the correct annotation of lipidomics data

**DOI:** 10.1038/s41467-021-24984-y

**Published:** 2021-08-06

**Authors:** Harald C. Köfeler, Thomas O. Eichmann, Robert Ahrends, John A. Bowden, Niklas Danne-Rasche, Edward A. Dennis, Maria Fedorova, William J. Griffiths, Xianlin Han, Jürgen Hartler, Michal Holčapek, Robert Jirásko, Jeremy P. Koelmel, Christer S. Ejsing, Gerhard Liebisch, Zhixu Ni, Valerie B. O’Donnell, Oswald Quehenberger, Dominik Schwudke, Andrej Shevchenko, Michael J. O. Wakelam, Markus R. Wenk, Denise Wolrab, Kim Ekroos

**Affiliations:** 1 Core Facility Mass Spectrometry and Lipidomics, ZMF, Medical University of Graz, Graz, Austria; 2 Institute for Molecular Biosciences, Karl-Franzens University of Graz, Graz, Austria; 3 Department for Analytical Chemistry, University of Vienna, Vienna, Austria; 4 Department of Physiological Sciences, College of Veterinary Medicine, University of Florida, Gainesville, FL USA; 5 University Duisburg Essen, Essen, Germany; 6 Department of Pharmacology, University of California, San Diego, CA USA; 7 Institute of Bioanalytical Chemistry, Faculty of Chemistry and Mineralogy, Universität Leipzig, Leipzig, Germany; 8 Center for Biotechnology and Biomedicine, Universität Leipzig, Leipzig, Germany; 9 Swansea Univerity Medical School, Swansea, UK; 10 Barshop Inst Longev & Aging Studies, Univ Texas Hlth Sci Ctr San Antonio, San Antonio, TX USA; 11 Institute of Pharmaceutical Sciences, University of Graz, Graz, Austria; 12 Department of Analytical Chemistry, Faculty of Chemical Technology, University of Pardubice, Pardubice, Czech Republic; 13 Department of Environmental Health Sciences, School of Public Health, Yale University, New Haven, CT USA; 14 Department of Biochemistry and Molecular Biology, VILLUM Center for Bioanalytical Sciences, University of Southern Denmark, Odense, Denmark; 15 Cell Biology and Biophysics Unit, European Molecular Biology Laboratory, Heidelberg, Germany; 16 Institute of Clinical Chemistry and Laboratory Medicine, University of Regensburg, Regensburg, Germany; 17 Systems Immunity Research Institute, Cardiff University, Cardiff, United Kingdom; 18 Bioanalytical Chemistry, Research Center Borstel, Borstel, Germany; 19 German Center for Infection Research, Borstel, Germany; 20 German Centre for Lung Research, Airway Research Center North, Borstel, Germany; 21 Max Planck Institute of Molecular Cell Biology and Genetics, Dresden, Germany; 22 Babraham Institute, Babraham Research Campus, Cambridge, UK; 23 Singapore Lipidomics Incubator (SLING), Department of Biochemistry, YLL School of Medicine, National University of Singapore, Singapore, Singapore; 24 Lipidomics Consulting Ltd., Esbo, Finland

**Keywords:** Lipidomics, Mass spectrometry, Data processing

**Arising from** M. Mann et al. *Nature Communications* 10.1038/s41467-019-14044-x (2020).

A recent publication from Vasilopoulou et al.^1^ reports on full lipidome profiling by a combination of trapped ion mobility spectrometry (TIMS), parallel accumulation serial fragmentation (PASEF) and nano HPLC^1^. While this represents an impressive technological advance with the potential to increase lipidome coverage and lower detection limits for individual lipids, the interpretation of the acquired spectra is a matter of concern. Specifically, the authors relied exclusively on software-assisted lipid assignments that were not confirmed by an independent inspection of matched spectra to recognize abundant structurally unique lipid fragments. Further, no attempts were made to correlate the retention times of identified species with available lipid standards, which constitutes the gold standard typically employed in lipidomics to reduce false-positive assignments. Manual inspection of the dataset performed by us suggested that the identification of at least 510 out of 1108 features reported as unique lipids would require additional experimental evidence. This, in turn, compromises the assignment of collision cross section (CCS) values for 1856 features, potentially misguiding other lipidomics laboratories that may use these CCS data for identifying lipids.

Automated lipid species annotation based on fragment ion mass spectra (MS^n^ spectra) faces three major challenges: (i) Isobaric or isomeric lipid species from different classes often yield similar fragments and cannot be unambigiously matched; (ii) the abundance of lipid fragments strongly depends on the experimental conditions^2^ which compromises their similarity to reference spectra; (iii) fragmentation of co-isolated precursors often originating from different classes yields highly convoluted spectra. Consequently, further, inspection is indispensable for spectra that were matched to lipid structures by software tools. Rule-based or decision tree-based approaches are more suitable for automated spectral annotation, such as lipid data analyzer (LDA)^2,3^, LipidHunter^4^, LipidXplorer^5^, LipidMatch^6^, and MS-DIAL^7^, to mention only a few common tools. These algorithms scout spectra for fragmentation patterns characteristic to each lipid class according to established fragmentation pathways and peak intensity relationships. Nonetheless, the key for correct unequivocal lipid species annotation lies in two other peculiarities of lipids that do not pertain to the interpretation of MS^n^ spectra: (a) lipids often form more than one adduct ion in electrospray ionization; (b) all chromatographic modes exhibit a regular retention behavior of lipids, for example, the equivalent carbon number (ECN) model used for reversed-phase chromatography^8–10^. While double bond position, geometry, and regioisomerism have only minor influence on lipid retention, typically lipid species only elute in the retention time range expected for their ECN. Correspondingly, the detection of several adducts (preferably in both ion modes) and compliance with the ECN model are important for the correct annotation of lipid species. Several software applications utilize the ECN model^2,11^. One example is the LDA tool, which uses unambiguously annotated lipid species to fit Eq. (1), where x and y correspond to the number of carbon atoms and double bonds, RT is the retention time and A through G are the parameters that are automatically fitted for each lipid class and chromatographic setup^2^:

1 $$RT(x,y)=A* (1-B* {x}^{-C})+D* {e}^{(-E \,*\, y+F\, *\, x)}+G$$

The application of rule-based approaches can reduce the number of false positives down to 1–10% (depending on the lipid class and the complexity of the sample), which facilitates high-throughput lipidomics studies. However, exclusively relying on annotations by a single software without additional means of validation often leads to unacceptably high rates of false positive identification. In high-throughput lipidomics, fully automated annotation of spectra requires better physicochemical models correlating molecular structures of lipids with their chromatographic retention and MS^n^ fragmentation.

In the publication from Vasilopoulou et al., 55 out of the reported 171 triacylglycerols (TG) do not follow the ECN model (Fig. 1a). The proportion of glycerophospholipids mismatching the linear retention time – carbon atom/-double bond number correlation is even higher. Specifically, 130 out of the reported 301 diacyl phosphatidylcholine (PC) species do not follow the ECN predictions (Fig. 1b). The confidence of such annotations is doubtful, even when their CCS values are similar to annotations that corroborate the ECN model.

**Fig. 1:**
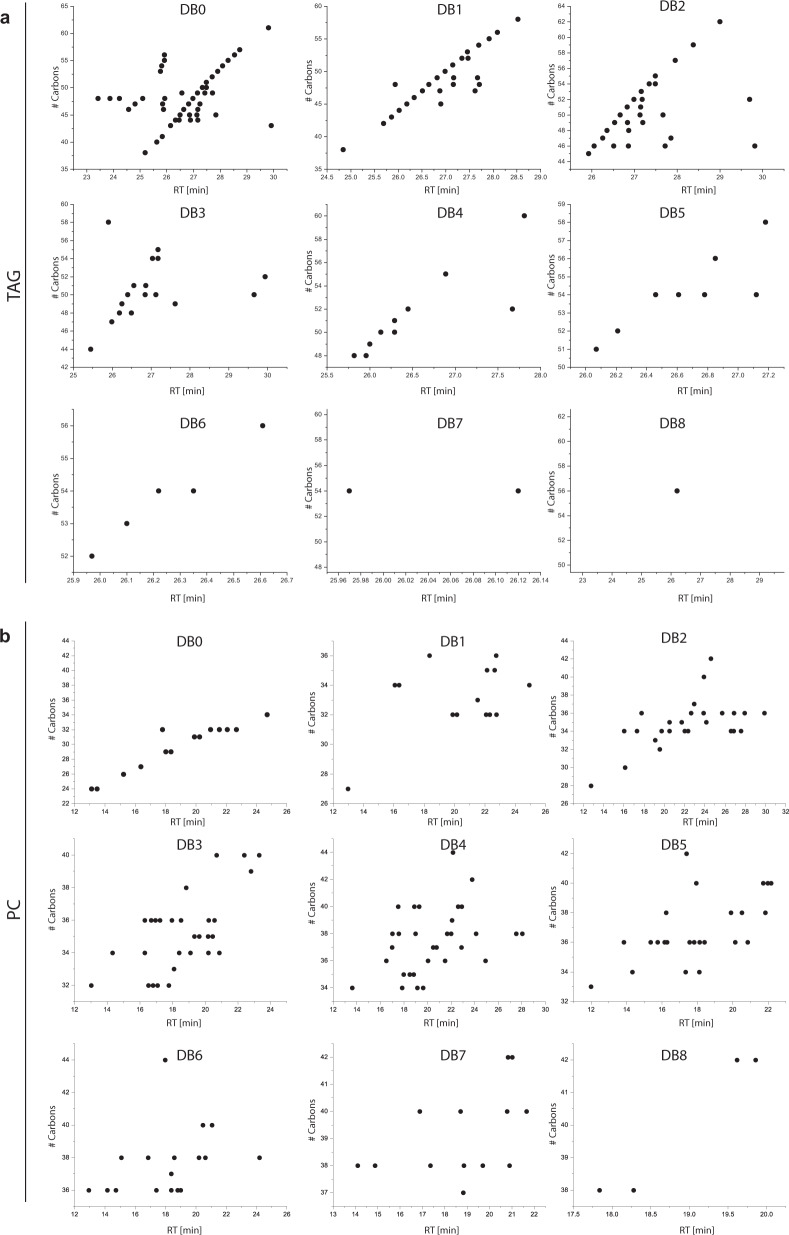
Plot of retention times versus number of fatty acyl carbons. Shown are data for (**a**) triacylglycerols and (**b**) diacyl phosphatidylcholines in plasma. DB0 through DB8 represent the cumulative number of double bonds in the fatty acyl chains. For each individual DB assignment the relationship should be close to linear on a C18 stationary phase colum.

The elution profiles of some reported lipids are unexpected for reversed phase chromatography. For example, three lipids annotated as DG 16:0/16:0 spread over the very large elution time range from 18.9 to 28.6 min, although only two lipids could be explained by regioisomerism. Typical retention time spreads do not exceed one or two minutes for this kind of chromatography; a 10 min time range is beyond reasonable explanation. Note that even more hydrophobic molecules having three fatty acid moieties like TG 16:0/16:0/16:0 eluted at 23.87 min—almost five minutes earlier than putative DG 16:0/16:0. Such examples are frequently encountered throughout the study from Vasilopoulou et al.

In several instances, identified lipids do not corroborate their chemical structure. While eight PC O-16:0_1:0 species were reported, only two *sn*-1/2 isomers (PC O-16:0/1:0 or PC O-1:0/16:0) can exist. Alternative structures for another six assignments comprising the same moieties (for example, including branched fatty alcohols or even more exotic *sn*-2/3 isomers) are in conflict with basic principles of lipid biosynthesis in mammals and must be validated by independent means, possibly including chemical synthesis of authentic molecules. Similarly, five chromatographic peaks annotated as CE 18:2 and four peaks annotated as cholesteryl 11-hydroperoxy-eicosatetraenoate would suggest more isomers for these lipid species than are likely based on their chemical structures. The elemental composition of recognized lipids must always match the *m/z* of their intact molecular ions within the method-dependent mass tolerance. A few more examples of questionable annotations based on incorrect mass assignments are presented in Supplementary Data 1.

Upon low-energy CID/HCD, lipid precursors produce relatively few abundant and highly informative fragments that enable unequivocal lipid class attribution and identification of fatty acid moieties. The identification of phospholipids by matching spectra with missing characteristic head group fragments (e.g., PC, sphingomyelin (SM) in positive or phosphatidylinositol (PI) in negative modes) or their neutral losses (*e.g*. phosphatidylethanolamine (PE) or phosphatidylserine (PS) in positive mode) should be disregarded^2,6^. In MS^2^ spectra in positive ion mode, five PC ([M+H]^+^) precursors produced no phosphocholine head group fragment (*m/z* 184.07) that are exceptionally abundant within a broad range of collision energies. The identification of only 6% of all PCs (28/437) relied upon the complete set of characteristic masses (e.g., exact masses of intact precursor; head group fragment in positive as well as carboxylate anion fragments of fatty acid moieties in negative ion modes). This is essential to distinguish them from abundant SM that overlap with isotopic peaks of PC and produce the same head group fragment *m/z* 184.07.

Similar problems are apparent in the identification of other lipid classes. For example, SM 16:1;O2/25:0 indicates a very unusual combination of a sphingosine backbone and an N-amidated fatty acid. However, its MS^2^ spectrum only confirms the presence of a phosphocholine head group (*m/z* 184.07) and, hence, cannot distinguish it from SM 18:1;O2/23:0—a common mammalian sphingomyelin. If alternative structures could not be unequivocally resolved by MS^2^, the corresponding precursors should be annotated by total number of carbon atoms and double bonds (e.g. SM 41:1;O2). We note that reporting the same feature or identified lipid by four different categories (Lipid name, Short name, LSI ID, and Lipid ID) might be confusing for some readers, especially if structure-specific annotation is not supported by MS^2^.

On several occasions lipid precursors were detected as uncommon adducts only, e.g. [M-CH_3_]^−^ for diacyl PI that have no methyl group to lose. The authors used a classical mobile phase containing 10 mM ammonium formate and formic acid. Therefore, in negative ion mode, formate molecular adducts of intact lipids are expected. However, with no specific explanation, 31% (10/32) diacylglycerols (DG), 21% (7/33) cholesteryl-esters (CE), 25% (1/4) ether-lysophosphatidylethanolamines (LPE) and 15% (11/72) of PE and ether-PE species were annotated as uncommon or unexpected adducts without detecting the corresponding prominent formate adduct. Nine PE and ether-PEs were only detected as acetate [M+AcO]^−^ adducts. However, even in 10 mM ammonium acetate buffer (which was not used), [M-H]^−^ but not [M+AcO]^−^ is the dominant molecular form for PE. Out of 437 PCs reported herein, 36 were detected as either redundant or unexpected adducts in negative ion mode. This warrants closer inspection of all available evidence before assigning them to unique lipids.

Lipidomes (including the plasma lipidome) are conserved molecular constellations and their quantification is an important means to validate the analytical concordance. Hence, the identification of very minor free sterols is highly surprising when no free cholesterol and none of its major metabolites were detected. Cholesterol is the most abundant single lipid in plasma whose molar concentration is more than 1000-fold higher than of any sterol reported by Vasilopoulou et al. Many sterols are present in plasma as multiple isomers, hence, without comparing CCS, retention times and fragmentation patterns to authentic standards, their identification is not reliable.

We underscore that problematic identifications are not limited to the examples discussed here. We believe that many of those uncertainties could have been sorted out by applying rational and commonly used requirements: the retention time of a proposed lipid should corroborate the retention time pattern of its lipid category/class; the elemental composition of identified species must match the accurate masses of their precursor ions; molecular adducts of intact molecular ions should be detected in the dominant form matching the mobile phase composition; and the detected fragments should be specific and corroborate the proposed lipid structure. Finally, structural annotation of each species (including identification of positional isomers) should match individual MS^2^ or (if available) MS^3^ spectra and cannot be unconditionally applied for the whole lipid class. When considering low abundant precursors or novel lipids, each spectrum should be re-inspected and, if possible, the proposed molecular structure should be confirmed by independent means. Although this could dramatically lower the number of lipid identifications, it vastly improves the data quality and integrity and ensures high biological relevance of the lipidome profile.

The lipidomics community worked over the last decades to improve the confidence of structural assignments and overall quality of lipidomics resources used as a reference in the field. One of the outcomes of these collaborations are guidelines for interpreting and reporting lipidomic data provided by the International Lipidomics Society (ILS), the Lipidomics Standards Initiative (LSI) and LIPID MAPS^12–14^. Analytical methods detecting very large numbers of lipids and metabolites are increasingly used by the biomedical community. However, we urge that these findings should be interpreted with healthy skepticism and analytical rigor, since CCS values such as those reported by Vasilopoulou et al. and incorporated in public resources (e.g., LIPID MAPS) will be widely used by other researchers.

## Supplementary information

Description of Additional Supplementary Files

Supplementary Data 1

## Data Availability

All relevant data are available from the authors.
