# Supplementary material for: Quality control requirements for the correct annotation of lipidomics data
Source: Nat Commun. 2021 Aug 6;12:4771. doi: 10.1038/s41467-021-24984-y (PMC8346590; doi:10.1038/s41467-021-24984-y)
Supplement: Supplementary file 1 — Description of Additional Supplementary Files [file 41467_2021_24984_MOESM1_ESM.docx]

**File Name: Supplementary Data 1**

Description: Selected examples of lipid IDs with annotation issues and suggestions for possible corrections.
